# Scale-Up Synthesis and In Vivo Anti-Tumor Activity of Curcumin Diethyl Disuccinate, an Ester Prodrug of Curcumin, in HepG2-Xenograft Mice

**DOI:** 10.3390/pharmaceutics11080373

**Published:** 2019-08-01

**Authors:** Chawanphat Muangnoi, Pahweenvaj Ratnatilaka Na Bhuket, Ponsiree Jithavech, Wisut Wichitnithad, Onsiri Srikun, Chakkrapan Nerungsi, Suthiluk Patumraj, Pornchai Rojsitthisak

**Affiliations:** 1 Pharmaceutical Chemistry and Natural Products Program, Faculty of Pharmaceutical Sciences, Chulalongkorn University, Bangkok 10330, Thailand; 2 Natural Products for Ageing and Chronic Diseases Research Unit, Chulalongkorn University, Bangkok 10330, Thailand; 3 Department of Analytical Development, Pharma Nueva Co. Ltd., Bangkok 10900, Thailand; 4 Pharmaceutical Ingredient Research Group, Research and Development Institute, The Government Pharmaceutical Organization, Bangkok 10400, Thailand; 5 Center of Excellence for Microcirculation, Department of Physiology, Faculty of Medicine, Chulalongkorn University, Bangkok 10300, Thailand; 6 Department of Food and Pharmaceutical Chemistry, Faculty of Pharmaceutical Sciences, Chulalongkorn University, Bangkok 10330, Thailand

**Keywords:** curcumin diethyl disuccinate, curcumin, scale-up synthesis, anti-tumor activity, HepG2-xenograft mice

## Abstract

Previously, we synthesized curcumin and a succinate ester prodrug of curcumin namely curcumin diethyl disuccinate (CurDD) in the lab scale, which yielded hundred milligrams to few grams of the compounds. CurDD was found to be more stable in a phosphate buffer pH 7.4 and exhibited better cytotoxicity against Caco-2 cells than curcumin. Here, the one-pot syntheses of curcumin and CurDD were scaled up to afford multigram quantities of both compounds for preclinical studies using a 10-L chemical reactor. The key steps for the synthesis of curcumin were the formation of boron-acetylacetone complex and the decomplexation of boron-curcumin complex. The synthesis of CurDD could be achieved via a one-step esterification between curcumin and succinic acid monoethyl ester chloride using 4-(*N*,*N*-dimethylamino)pyridine as a catalyst. The synthesized curcumin and CurDD were then investigated and compared for an anti-tumor activity in HepG2-xenograft mice. CurDD could reduce the tumor growth in HepG2-xenograft mice better than curcumin. CurDD also exerted the stronger inhibition on VEGF secretion, COX-2 and Bcl-2 expression and induced higher Bax expression in comparison with curcumin. The results suggest that CurDD is a promising prodrug of curcumin and has a potential to be further developed as a therapeutic agent or an adjuvant for the treatment of hepatocellular carcinoma.

## 1. Introduction

**Figure 1:**
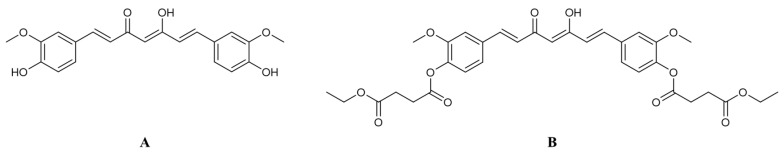
Chemical structures of (**A**) curcumin and (**B**) curcumin diethyl disuccinate.

Curcumin (Figure 1A) is a polyphenolic substance found in turmeric (*Curcuma longa* L.) with numerous pharmacological activities including anti-inflammation [1,2], anti-oxidation [3] and anti-angiogenesis [4]. It also has been investigated for anti-cancer activity against several cancer types such as breast, prostate, lung, colon and liver cancers [5]. Curcumin induces apoptosis in cancer cells via the mitochondria-mediated pathway [6,7,8]. It also suppresses the expression of the vascular endothelium growth factor (VEGF) and cyclooxygenase-2 (COX-2) [9]. Even though curcumin has promising anti-cancer effects, the use of curcumin in cancer chemotherapy is hindered by the poor oral bioavailability due to the chemical instability and extensive first-pass metabolism [10].

Prodrug design has been utilized to improve undesired physicochemical, biopharmaceutical and pharmacokinetic properties of several drugs and phytochemicals. Previously, we developed a succinate ester prodrug of curcumin namely curcumin diethyl disuccinate (CurDD, Figure 1B) to improve chemical stability and reduce phase II metabolism of curcumin. CurDD demonstrated better chemical stability in phosphate buffer pH 7.4 in comparison with curcumin [11] and exhibited good antinociceptive activities in pain models [12]. An in vivo pharmacokinetic study of CurDD in rats showed that oral administration of CurDD at a dose of 40 mg/kg gave the similar plasma level of curcumin to that of curcumin given orally at the same dose [13]. This evidence implies that CurDD improved the oral bioavailability of curcumin because CurDD has a molecular weight (MW) approximately 1.7 times higher than curcumin (MW 624 compared to 368). In addition, CurDD can be converted to curcumin in plasma rapidly in vitro and after intravenous administration to rats, suggesting that the administration of CurDD may have the similar onset time in exerting pharmacological actions compared with curcumin. [14,15]. Therefore, CurDD is a potential curcumin prodrug that can be further developed for clinical applications.

Hepatocellular carcinoma (HCC) is the most common malignant tumor that accounts for nearly 80% of all liver cancer cases [16]. HCC is a leading cause of cancer morbidity and mortality in men and women throughout developing countries [17]. It has been shown that the prolonged overproduction of reactive oxygen species and reactive nitrogen species induced by toxic substances or hepatitis B virus infections leads to chronic inflammation liver. The chronic damage of liver cells, DNA, and activation of hepatic stellate cell lead to the development of malignant tumor and HCC [18,19]. The conventional cancer treatments including surgical resection, radiotherapy, and chemotherapy have been found to be ineffective or only minimally effective in patients with unresectable HCC and cause toxicity to normal cells resulting in serious side effects [16]. The use of antioxidative substances may alleviate or delay the progression of HCC. Therefore, the development of antioxidants as alternative chemotherapeutic agents or adjuvants with acceptable efficacy and low toxicity would be of benefit to the treatment of HCC.

Curcumin has been demonstrated to have cytotoxicity against HepG2 cells [20,21] and the anti-hepatocellular carcinoma effect in vivo [4,22]. However, the chemical instability of curcumin limits itself from being developed as a chemotherapeutic agent. As mentioned earlier, CurDD is more stable than curcumin and has a potential to be further developed for the treatment of HCC. In order to expedite the development of CurDD for applications in cancer therapy, a practical large-scale synthesis method is required to have sufficient materials for preclinical studies. Here, we describe synthesis procedures for the large-scale production of curcumin and CurDD and their inhibitory action on tumor growth in a HepG2-xenograft mice model. We found that the scale-up methods reported here gave good yields and high purity of curcumin and CurDD raw materials and CurDD could inhibit the growth of tumor in the higher extent than curcumin.

## 2. Materials and Methods

### 2.1. Scale-Up Methods for Curcumin and CurDD Syntheses

Commercial grade ethyl acetate (≥98%), hexanes (≥98%), dichloromethane (≥99%) and ethanol (≥96%) were obtained from RCI Labscan (Bangkok, Thailand) and used as received from commercial suppliers without further purification. Boron oxide (≥98%), acetyl acetone (≥99%), vanillin (99%), tributyl borate (≥99%), 4-(*N*,*N*-dimethylamino)pyridine (DMAP) and succinic acid monoethyl ester chloride (95%) were purchased from Sigma-Aldrich (St. Louis, MO, USA). *n*-Butylamine (99.5%) was procured from ACROS Organics (Geel, Belgium ). *n*-Octanol was obtained from Merck (Darmstadt, Germany). Water was produced in-house using a Milli-Q water purification system (Millipore S.A.S, Molsheim, France)

#### 2.1.1. Synthesis of Curcumin

A 17.5 g (0.25 mol) of boron oxide, 0.05 L (0.50 mol) of acetylacetone and 0.5 L of ethyl acetate were mixed in a round bottom flask. The mixture was stirred at 50 °C, 100 rpm for 1 h. After a white boron-acetylacetone complex was formed, the mixture was cooled to 25 °C and transferred to a 10-L MINIPILOT GO47472 chemical reactor (Buchiglassuster, Switzerland). A 150 g (1 mol) of vanillin, 0.5 L (1.8 mol) of tributyl borate and 1.5 L of ethyl acetate were added to the reaction mixture and stirred at 200 rpm for 20 min. Then, a 0.1 L of 25% *n*-butylamine in ethyl acetate (0.25 mol of *n*-butylamine) was added dropwise to the reaction mixture over 1 h. The reaction mixture was continuously stirred at 25 °C, 200 rpm. After stirring for 20 h, 2.5 L of 1 M HCl (2.5 mol) was added to the reaction mixture and stirred for 30 min. Then, the stirring was stopped to allow the separation between an organic layer and an aqueous layer to take place. The organic layer was collected, and the aqueous layer was extracted with 2.5 L ethyl acetate twice. The combined organic layer was washed with 1.5 L of 5% NaCl solution twice. All aqueous layers were discarded. The extraction process was performed in the 10-L chemical reactor and the mixture of the aqueous layer and ethyl acetate was stirred at 25 °C, 200 rpm for 10 min. The combined organic layer was stirred at 60 °C, 200 rpm under reduced pressure until the amount of the combined organic phase was reduced to approximately 4 L. Subsequently, a 2.5 L of hexanes was slowly added to the concentrated ethyl acetate phase. The mixture between hexanes and the concentrated ethyl acetate phase was stirred at 25 °C, 150 rpm under normal atmospheric pressure to obtain a suspension of curcumin. The suspension was filtered to collect orange solids. The orange solid was dried in a hot air oven at 45 °C (118.02 g, %yield = 64.1). The dried solids were analyzed by proton nuclear magnetic resonance (^1^H-NMR), high-resolution mass spectrometric (HRMS) and ultra-high performance liquid chromatographic (UHPLC) techniques. ^1^H-NMR signals (300 MHz, CDCl_3_) at δ 7.61 (d, *J* = 15.8 Hz, 2H, H1 and H7), 7.19–7.04 (m, 4H, H5′, H5″, H6′ and H6″ protons,), 6.95 (d, *J* = 8.2 Hz, 2H, H2′ and H2″), 6.49 (d, *J* = 15.8 Hz, 2H, H2 and H6), 5.84 (d, *J* = 14.4 Hz, 1H, H4), 3.99 (s, 6H, OCH_3_,). HRMS (ESI) calculated for C_21_H_21_O_6_ [M+H]^+^ 369.1333; observed 369.1331. HRMS (ESI) calculated for C_21_H_20_O_6_Na [M+Na]^+^ 391.1152; observed 391.1142. Chromatographic purity 100.0% by UHPLC (λ = 425 nm). 

#### 2.1.2. Synthesis of CurDD

A 50 g (0.14 mol) of curcumin was dissolved in 2.5 L of dichloromethane and a mixture was stirred at 25 °C, 100 rpm for 30 min in a 10-L chemical reactor. A 41.5 g (0.34 mol) of DMAP was added to the solution and stirred at 25 °C, 100 rpm for 20 min. Then, a 50 mL (0.31 mol) of succinic acid monoethyl ester chloride was added dropwise to the solution. Then, the propeller speed was increased to 200 rpm and this stirring speed was used in the subsequent steps. The completeness of the reaction was monitored by a silica gel 60 F254 thin-layer chromatography using 2:3:1 (*v*/*v*) of hexane:ethylacetate:methanol as a mobile phase. After stirring for 2 h, the solution was acidified with a 1.5 L of 0.1 M HCl (0.15 mol) for 2 min. The aqueous HCl layer was discarded, and an organic phase was mixed with a 1.5 L of 1% sodium bicarbonate (NaHCO_3_). The mixture was stirred for another 2 min. The aqueous NaHCO_3_ mixture was removed, and the organic layer was washed with a 1.5 L of deionized water twice. The residual water was eliminated using a 1.5 L of saturated NaCl solution. The organic layer was collected into a round-bottom flask and evaporated using a rotary evaporator yielding amber viscous liquid. A 200 mL of ethanol was added to the liquid and the mixture was stirred at room temperature until yellow solids were obtained. The yellow solids were heated in a hot air oven at 45 °C until no weight change (84.2 g, %yield = 99). The dried solids were analyzed by ^1^H-NMR, HRMS and UHPLC. ^1^H-NMR (300 MHz, CDCl3) spectrum shows signals at δ 7.63 (d, *J* = 15.8 Hz, 2H, H1 and H7), 7.24–7.04 (m, 6H, Ph), 6.58 (d, *J* = 15.8 Hz, 2H, H2 and H6), 5.87 (s, 1H, H4), 4.20 (q, *J* = 7.1 Hz, 4H, H11′ and H11″ protons,), 3.89 (s, 6H, OCH_3_), 2.95 (t, *J* = 6.9 Hz, 4H, H8′ and H8″), 2.76 (t, *J* = 6.9 Hz, 4H, H9′ and H9″), 1.29 (t, *J* = 7.1 Hz, 6H, C12′ and C12″). HRMS (ESI) calculated for C_33_H_37_O_12_ [M′+H]^+^ 625.2280; observed 625.2287. HRMS (ESI) calculated for C_33_H_36_O_12_Na [M′+Na]^+^ 647.2099; observed 647.2133. Chromatographic purity 99.8% by UHPLC (λ = 400 nm).

### 2.2. Determination of Physical and Chemical Properties of Curcumin and CurDD Raw Materials

#### 2.2.1. Solubility of Curcumin and CurDD

Water solubility of curcumin and CurDD was determined using the shake flask method as per a guideline of the Organization for Economic Cooperation and Development (OECD) [23]. A 10 mg of curcumin or CurDD was added to a flask. A 1 mL of water was added to the flasks containing curcumin or CurDD and the flasks were capped. The samples were continuously shaken at 100 rpm, at 25 °C for 24 h. Then, the samples were centrifuged and supernatants were collected for the HPLC analysis using the method previously described [11]. The experiment was performed in triplicate. The obtained water solubility of CurDD was compared to that of curcumin. The water solubility of curcumin was in agreement with previously reported [24].

#### 2.2.2. Partition Coefficient

The partition coefficients (P_o/w_) of curcumin and CurDD were evaluated using the shake-flask method of OECD [25]. *n*-Octanol was saturated with water and vice versa prior to starting the experiment. Curcumin and CurDD were separately dissolved in test vessels containing different *n*-octanol-to-water ratios of 1:1, 2:1 and 1:2. The samples were shaken horizontally 100 times in 5 min at 25 ± 1 °C using a mechanical shaker. Then, the two phases were separated by centrifugation at 4000 rpm at 25 °C for 10 min. The concentrations of curcumin or CurDD in each phase were determined by HPLC [11]. The P_o/w_ value was calculated based on data obtained from three duplicate runs of the different *n*-octanol-to-water ratios. The P_o/w_ values were expressed as the log P_o/w_. The log P_o/w_ values of curcumin and CurDD were calculated from the mean of the six log P_o/w_ values from individual samples.

#### 2.2.3. Determination of Particle Size Distribution

Particle size distribution of curcumin and CurDD raw materials was determined by a laser diffraction technique. The laser diffraction was performed on a Mastersizer 3000 with Hydro EV Flexible volume wet dispersion (Malvern Instruments, Worcestershire, UK). The particle size distributions of curcumin and CurDD were calculated using the Mastersizer 3000 software (version 5.54). The experiment was performed in triplicates.

#### 2.2.4. X-Ray Diffraction

Powder X-ray diffraction (PXRD) was performed to determine the crystallinity of curcumin and CurDD using a D2 PHASER benchtop diffractometer (Bruker, WI, USA) with Cu Kα radiation (λ = 1.5418 Å for combined K_α1_ and K_α2_). A 1 g of curcumin or CurDD was spread on a glass plate. The diffraction angle, the scan rate, the voltage and the current of the generator for recording XRD patterns were set at 5.0 to 35.0°, 4.0°/min, 40 kV and 15 mA, respectively.

#### 2.2.5. Melting Point Measurement

Melting points of curcumin and CurDD were determined by the differential scanning calorimetric (DSC) technique. DSC experiments were performed with a DSC 8000 instrument (Perkin Elmer, city, MA, USA). A 1–2 g of curcumin or CurDD was placed in open pans for melting point determination. All DSC scans were performed at the rate of 10 °C/min for both heating and cooling. During all the measurements, the calorimeter head was flushed with highly pure nitrogen gas.

### 2.3. Animals

The experiments were performed in male BALB/c-nude mice (20–25 g) purchased from Nomura Siam International, Thailand. The nude mice were housed in an animal facility of the Faculty of Medicine, Chulalongkorn University under standard conditions at 25 ± 2 °C and 50–60% of humidity under a 12 h light/dark cycle. The mice were kept under laboratory conditions for one week prior to the start of the experiment and allowed food and water *ad libitum*. All animal experiments were conducted according to the Ethical Guidelines for the Uses of Animals by The National Research Council of Thailand (1999). The experimental protocol (protocol number 019/2559) was approved by the Institutional Animal Care and Use Committee of the Faculty of Medicine, Chulalongkorn University, Bangkok, Thailand (approval number 01/2560; approved on 1 Mar 2017). 

### 2.4. Implantation of Tumor Cells

To generate tumors, the nude mice were injected with 100 µL of a single cell suspension containing 5 × 10^6^ of HepG2 cells in DMEM into the subcutaneous layer of dorsal skin in mice. The tumor volume (V) was calculated using its mean diameter as measured with Vernier calipers every three days and then using the formula, v = 0.5 × a × b^2^ (where a and b are the shortest and the longest diameters, respectively.) In addition, the body weight of nude mice was determined every three days similar to the tumor volume.

### 2.5. Curcumin and CurDD Treatments

Doses of curcumin were orally administered at 300 (low dose) and 3000 (high and effective dose) mg/kg bodyweight (BW) according to the previously published study by Yoysungnoen et al. [4]. Since CurDD is a prodrug of curcumin, we therefore calculated doses of CurDD to have the equivalent amount of curcumin based on the molecular weights of CurDD and curcumin at 624.6 and 368.4, respectively. The doses of CurDD were administered at 500 and 5000 mg/kg BW corresponding to those of curcumin at 300 and 3000 mg/kg BW, respectively. The nude mice received treatments and were divided into ten groups as follows (*n* = 8/group): (1) Normal mice (Control), (2) curcumin 300 mg/kg BW treatment (Cur300), (3) curcumin 3000 mg/kg BW treatment (Cur3000), (4) CurDD 500 mg/kg BW treatment (CurDD 500), (5) CurDD 5000 mg/kg BW treatment (CurDD 5000), (6) HepG2-induced tumor mice (HepG2), (7) HepG2-induced tumor mice with curcumin 300 mg/kg BW treatment (HepG2-Cur300), (8) HepG2-induced tumor mice with curcumin 3000 mg/kg BW treatment (HepG2-Cur3000), (9) HepG2-induced tumor mice with CurDD 500 mg/kg BW treatment (HepG2-CurDD500), and (10) HepG2-induced tumor mice with CurDD 5000 mg/kg BW treatment (HepG2-CurDD5000). Curcumin and CurDD treatments were started at day seven after the injection of HepG2 cells. Nude mice treated daily orally with 200 µL of curcumin (300 and 3000 mg/kg BW) or CurDD (500 and 5000 mg/kg BW) dissolved in corn oil for three weeks. The control and HepG2 groups received the vehicle (corn oil).

### 2.6. Aspartate Aminotransferase, Alanine Aminotransferase and VEGF Determination

Blood samples were obtained by a cardiac puncture and collected in a microcentrifuge tube. After allowing to clot for half an hour, the blood samples were centrifuged at 5000 rpm for 10 min. Resulting sera were separated and stored at −20 °C until analysis. The levels of aspartate aminotransferase (AST) and alanine aminotransferase (ALT) were performed using AST and ALT assay kits (Biocompare, South San Francisco, city, CA, USA). The levels of VEGF were determined by the VEGF ELISA kit (Sigma-Aldrich, St. Louis, city, MO, USA).

### 2.7. Western Blot Analysis for Tumor Tissues

Tumor tissues were excised from the dorsal skin-fold chamber and washed with cold PBS. The tissue samples were cut with medium-sized scissors on ice block, containing a tissue lysis buffer (RIPA buffer supplemented with protease and phosphatase inhibitors). Then, the tissue fragments were homogenized in an ice bath using a homogenizer at 10,000 rpm for 5 min. To remove the tissue debris, homogenates were centrifuged at 3500 rpm 4 °C for 40 min. The supernatant was collected and aliquoted into sterile microcentrifuge tubes, kept at −80 °C until it was used. Samples containing 50 μg of protein were added into 10% SDS-PAGE, transferred to a pure nitrocellulose membrane (Amersham™ Protran^®^, Sigma Aldrich), and blocked with 5% dry milk. The membrane was incubated with antibodies against Bax (1:1000), Bcl-2 (1:1000), COX-2 (1:1000) or β-actin (1:5000) at 4 °C overnight. Then, membranes were washed with TBST and incubated with species-specific HRP conjugated secondary antibody reacted with Super Signal solution (Endogen Inc., Rockford, IL, USA) for 2 min. The membrane was detected band density with chemiluminescence documentation (ImageQuant LAS 4000, GE Healthcare Bio-Sciences, Umea, Sweden) and then, stripped off the bound antibody and re-probed with anti-β actin antibody to confirm the equal loading of protein. Results were expressed as a relative ratio of band intensity of the target proteins and β-actin.

### 2.8. Statistical Analysis

All experiments were performed at least in four replicates unless indicated otherwise. Statistical analysis was performed using the SPSS version 19.0 (SPSS Inc., Chicago, IL, USA). Data are presented as mean ± standard deviation (SD). Means were compared by the one-way analysis of variance (ANOVA) with Scheffe and Duncan’s post hoc test. Differences were considered significantly at *p* < 0.05.

## 3. Results and Discussion

### 3.1. Synthesis of Curcumin and CurDD

**Scheme 1:**
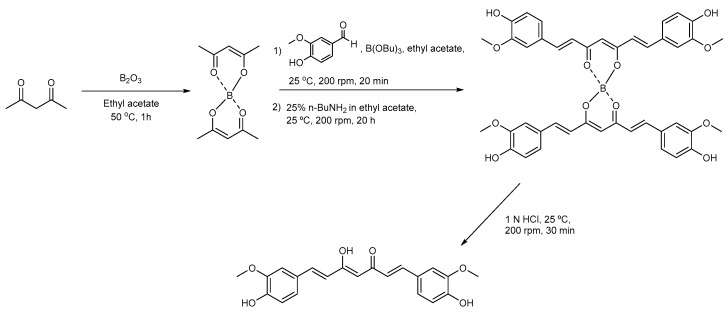
Synthetic route of curcumin.

In this work, the synthesis methods of curcumin and CurDD were modified and scaled up 50 and 250 times, respectively, to the previously published methods [11]. As shown in Scheme 1, the first step in the synthesis of curcumin is the formation of the boron-acetylacetone complex. The complexation between boron and acetylacetone prevents the Knovenagel condensation at C3 of acetylacetone [11]. The formation of this complex could be observed as the development of a milky mixture within 1 h. Upon the addition of *n*-butylamine, an α-proton of a methyl group of boron-acetylacetone complex was abstracted to generate a carbanion which subsequently underwent aldol condensation with an aldehyde group of vanillin. Tributyl borate was used to eliminate water formed during the reaction, hence facilitating the condensation reaction. The resultant product was the boron-curcumin complex with two molecules of curcumin bound to one boron atom. The boron-curcumin complex was then dissociated by hydrochloric acid (HCl). The complexation between the boron and acetylacetone and the decomplexation of the boron-curcumin complex are essential steps to yield curcumin. The use of ethyl acetate as a reaction solvent provided suitable solubility for reactants and products and eased the removal of the aqueous phases from the chemical reactor during extraction. We used hexanes as an antisolvent to precipitate curcumin from the ethyl acetate phase because the crystallization of curcumin using methanol described in our previous work [11] was not practical for purifying the compound in the chemical reactor. The obtained curcumin was then used for the synthesis of CurDD and in vivo evaluation.

**Scheme 2:**
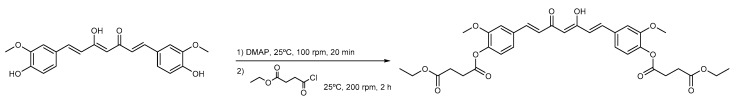
Synthesis route of curcumin diethyl disuccinate.

The synthesis route for CurDD is shown in Scheme 2. The esterification of curcumin with succinic acid monoethyl ester chloride was performed in dichloromethane, which solubilized all reactants and products. It is worth noting that DMAP hydrochloride generated in the reaction was not precipitated out from the reaction solution. Unlike dichloromethane, in the preliminary study, we performed the reaction using ethyl acetate as a reaction solvent and observed the precipitation of the DMAP hydrochloride as white solids, which could prevent the completeness of the reaction by occluding the reactants within the precipitates. Dichloromethane was therefore chosen as the reaction solvent in this study. The removal of DMAP was achieved by the addition of an aqueous HCl to the reaction solution. All DMAP was converted to the DMAP hydrochloride and subsequently partitioned into the acidic aqueous phase, which is 0.1 N HCl. The subsequent treatment of 1% NaHCO_3_ was performed to neutralize the residual HCl in the dichloromethane layer. After the evaporation of dichloromethane, the reaction product became an oily amber liquid. The solidification of the reaction product was achieved after stirring the oily liquid with ethanol.

The synthesized curcumin and CurDD were structurally confirmed by ^1^H-NMR (Supplementary Figures S1 and S2) and HRMS (Supplementary Figures S3 and S4) techniques and the results were in agreement with our previous data [11]. The chromatographic purity results from the UHPLC analysis of the synthesized curcumin and CurDD are shown in Supplementary Figures S5 and S6, respectively. Curcumin had a retention time of 1.14 min with the chromatographic purity of 100.0% (Supplementary Figure S5). The UHPLC chromatogram of CurDD (Supplementary Figure S6) shows a major peak at 5.32 min, which is CurDD, and a minor peak at 5.14 min, which is expected to be another tautomer of CurDD similar to our previous reports [14,15]. Combining the peak areas of these two peaks, the chromatographic purity was found to be 99.8%. In the future work, a method for the synthesis of CurDD at an industrial scale should be developed and optimized for extensive preclinical and clinical studies. Additionally, a stability-indicating method and other assays, e.g., residual solvent, organic contaminations and sulfated ash should be defined for the quality control of CurDD raw materials.

### 3.2. Physical and Chemical Properties of Curcumin and CurDD

Various physical and chemical properties including water solubility, partition coefficient, crystallinity, and particle size distribution of drug molecules may affect in vivo pharmacological activities. In this study, the solubility of CurDD in water at 25 °C was found at 0.059 µg/mL which was slightly less than that of curcumin at 0.068 µg/mL [24]. Unlike curcumin, the phenolic hydroxyl groups of CurDD were esterified with ethyl succinyl groups that contribute to the slight difference in the water solubility of curcumin and CurDD. The partition coefficients of curcumin and CurDD are in agreement with the findings on their water solubility. The log P_o/w_ value of CurDD and curcumin was 2.55 and 2.19, respectively. The P_o/w_ of CurDD is approximately 2.3 times higher than that of curcumin, indicating that the introduction of two ethyl succinyl groups greatly increase the lipophilicity of CurDD compared with curcumin.

PXRD was performed to evaluate the crystallinity of the synthesized curcumin and CurDD. As shown in Supplementary Figures S7 and S8, the presence of peaks in the PXRD spectra of curcumin and CurDD, respectively, indicated that curcumin and CurDD powders were in a crystal form. However, the particle size distribution between the powders of curcumin and CurDD were different. The synthesized CurDD had a broad range of particle size ranging from 0.5 µm to 300 µm (Supplementary Figure S9) while the synthesized curcumin had a narrower range of particle size ranging from 0.5 µm to 30 µm (Supplementary Figure S10). This noticeable dissimilarity could be derived from the different crystallization methods. Curcumin was solidified by an anti-solvent using the chemical reactor with specified paddle speed whereas CurDD was crystalized by ethanol using a round-bottom flask. Melting points of the prepared curcumin and CurDD were in the range of 185–186 °C and 95–96 °C, respectively, which were consistent with our previous findings [11].

### 3.3. Effects of Curcumin and CurDD on Body Weight, AST and ALT Activities

**Figure 2:**
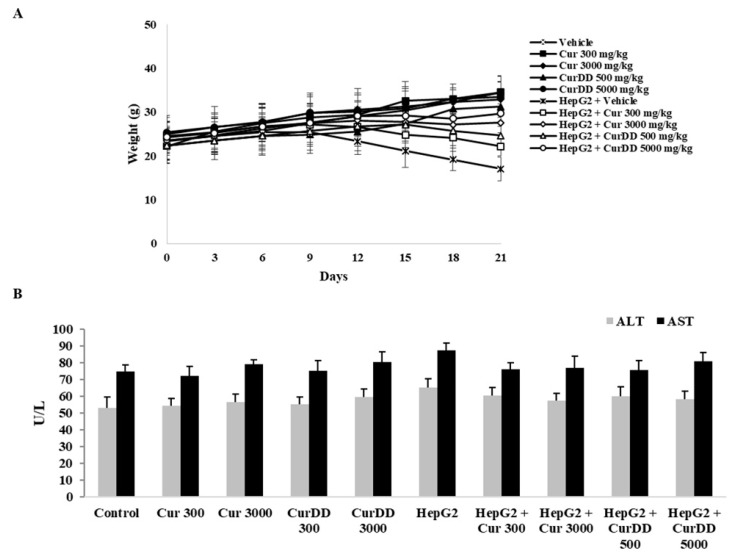
Effects of curcumin and CurDD on (**A**) body weight, (**B**) AST and ALT activities of HepG2- transplanted tumors in nude mice and treatment groups at 21 days after treatment. Corn oil was used as a vehicle for curcumin and CurDD. (Data are presented as mean ± SD (*n* = 8)).

The mean body weight of the nude mice in the treatment groups was slightly smaller than that of the control group throughout the study period but not statistically significant (*p* < 0.05) (Figure 2A). The mean body weight of the HepG2-induced tumor mice group was significantly decreased compared to the control group (*p* < 0.05). It was also noted that the HepG2-induced tumor had an effect on the mean body weight. The AST and ALT activities of nude mice in the test groups did not significantly differ from those of the control group throughout the study period (*p* < 0.05) (Figure 2B), indicating that curcumin, CurDD and HepG2 implantation did not affect the AST and ALT activities. It should be noted that the level of serum AST and ALT in rats receiving curcumin and CurDD were not different from those of the control group even at high doses of curcumin and CurDD (3000 and 5000 mg/kg, respectively), These data further indicate that curcumin and CurDD do not lead to liver toxicity and can therefore be used safely.

### 3.4. Inhibition of Tumor Growth by Curcumin and CurDD

**Figure 3:**
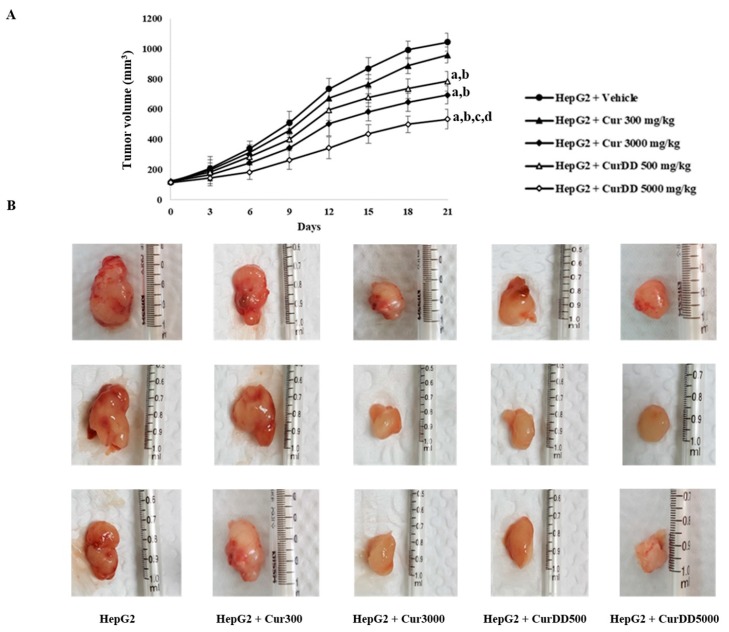
Effects of curcumin and CurDD on (**A**) tumor volume and (**B**) tumor size of HepG2-transplanted tumors in nude mice and in treatment groups at 21 days after treatment. Corn oil was used as a vehicle for curcumin and CurDD. (Data are presented as mean ± SD (*n* = 8); ^a^ *p* < 0.05 compared to HepG2 group+Vehicle, ^b^ *p* < 0.05 compared to HepG2+Cur 300 mg/kg group, ^c^ *p* < 0.05 compared to HepG2+Cur 3000 mg/kg group, and ^d^ *p* < 0.05 compared to HepG2+CurDD 500 mg/kg group).

One week after inoculation of the nude mice with HepG2 cells, a tumor bud could be observed and enlarged continuously over time as shown in Figure 3. The tumor volume of Cur300 group was not significantly different compared to the HepG2 group while the tumor volumes of the Cur3000, CurDD500 and CurDD5000 groups were significantly smaller than the tumor volume of the HepG2 group (*p* < 0.05) at day 15 after treatment. At day 21 after treatment, the tumor volumes of the CurDD500 and Cur5000 groups were significantly smaller than the tumor volume of the HepG2 group (*p* < 0.05). Additionally, the tumor volumes of the CurDD500 and Cur5000 groups were significantly less than that of the Cur300 and Cur3000 groups (*p* < 0.05), respectively. Interestingly, the oral administered CurDD at 500 mg/kg gave the comparable anti-tumor effect to the administration of curcumin at 3000 mg/kg, suggesting that CurDD could enhance the oral bioavailability of curcumin. Further investigation to obtain the similar effects of CurDD at 500 mg/kg may be performed using lower doses of CurDD, allowing the practical doses for future clinical studies.

### 3.5. Effects of Curcumin and CurDD on VEGF Secretion, COX-2, Bax and Bcl-2 Expression

**Figure 4:**
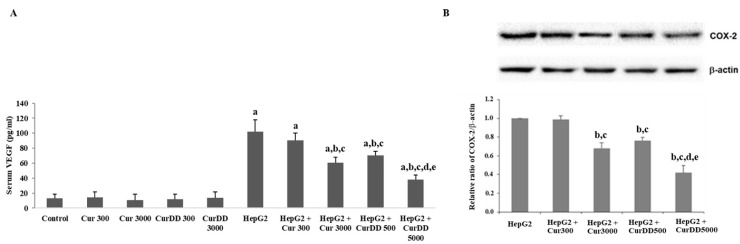
Effects of curcumin and CurDD on (**A**) serum vascular endothelium growth factor (VEGF) secretion and (**B**) cyclooxygenase-2 (COX-2) expression from tumor tissue of a HepG2-transplanted tumor in nude mice and in the treatment groups at 21 days after treatment. (Data are presented as means ± SD (*n* = 8); ^a^ *p* < 0.05 compared to control group, ^b^ *p* < 0.05 compared to HepG2 group, ^c^ *p* < 0.05 compared to HepG2+Cur 300 mg/kg group, ^d^ *p* < 0.05 compared to HepG2+Cur 3000 mg/kg group, and ^e^ *p* < 0.05 compared to HepG2+CurDD 500 mg/kg group).

Figure 4 shows the results of the quantification of VEGF secretion and COX-2 expression in HepG2-induced tumor mice in comparison with the control group. The treatment with Cur3000, CurDD500 and CurDD5000 significantly decreased the VEGF secretion and COX-2 expression in comparison with the HepG2-induced tumor mice group (*p* < 0.05). It is well known that VEGF plays an important role in the angiogenesis of cancerous tissues while COX-2 appears to induce angiogenesis in various types of tumors [26,27]. The suppression of the mitogen-activated protein kinase (MAPK) and nuclear factor kappa B (NF-κB) signaling pathway might be the underlying mechanism for the anti-tumor activity of CurDD and curcumin since this signaling pathway regulates VEGF secretion and COX-2 expression [28,29]. The anti-angiogenesis effects of curcumin and CurDD led to the apoptotic induction of the tumor via the mitochondria-mediated caspase pathway as suggested by the activation of caspase-3 and -9, the increase of the pro-apoptotic protein Bax expression and the downregulation of anti-apoptotic Bcl-2 protein.

**Figure 5:**
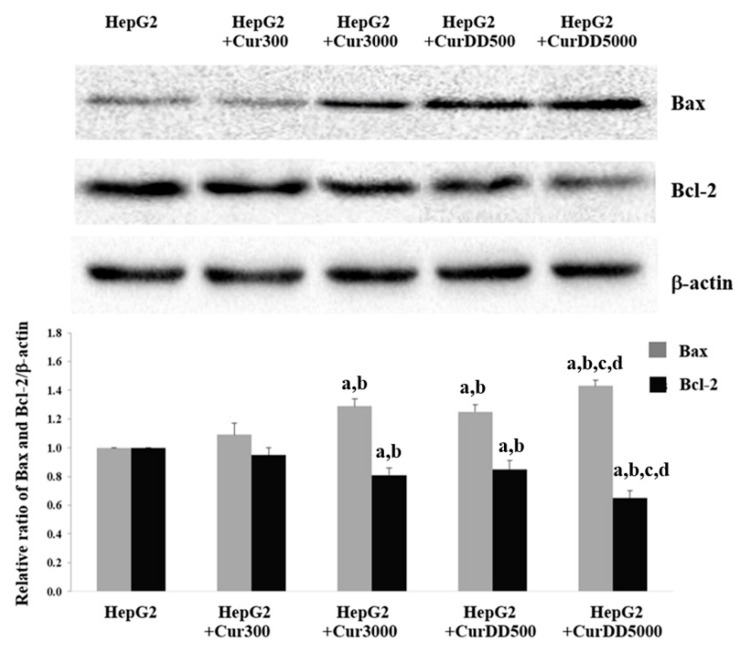
Effects of curcumin and CurDD on Bax and Bcl-2 expression from tumor tissue from tumor tissue of HepG2-transplanted tumor in nude mice and in the treatment groups at 21 days after treatment. (Data are presented as means ± SD (*n* = 8); ^a^ *p* < 0.05 compared to HepG2 group, ^b^ *p* < 0.05 compared to HepG2+Cur 300 mg/kg group, ^c^ *p* < 0.05 compared to HepG2+Cur 3000 mg/kg group, and ^d^ *p* < 0.05 compared to HepG2+CurDD 500 mg/kg group).

As shown in Figure 5, the expression of Bax protein increased by 25%, 20% and 40% after the treatment of Cur3000, CurDD500 and CurDD5000 for 21 days, respectively, in HepG2-induced tumor mice. In contrast, the treatment of Cur3000, CurDD500 and CurDD5000 in HepG2-induced tumor mice decreased the Bcl-2 expression by 20%, 15% and 35%, respectively. The suppressive effect of the CurDD was significantly higher than that of curcumin (*p* < 0.05). These results indicated that CurDD induced apoptosis in the HepG2-induced tumor mice better than curcumin. In addition, CurDD increased and decreased the expression of Bax and Bcl-2 proteins, respectively, to a higher extent than curcumin.

## 4. Conclusions

Large-scale synthesis methods for curcumin and CurDD were developed to produce sufficient amounts of both compounds for preclinical studies. The methods reported were modified from our lab-scale methods to enable the scaling up production. Due to their simple steps of production, the methods have a potential for further multikilogram-scale production. This study also shows that CurDD could enhance the anti-tumor activity of curcumin in the HepG2 xenograft mice. CurDD could inhibit or delay the growth of tumor in the higher extent than curcumin as demonstrated from the western blot analyses for VEGF secretion, COX-2, Bax and Bcl-2 expression. The results suggest that CurDD is a promising prodrug of curcumin and has the potential to be extensively investigated as a therapeutic agent or an adjuvant for the treatment of HCC in additional preclinical and clinical studies.

## Supplementary Materials

The following are available online at https://www.mdpi.com/1999-4923/11/8/373/s1, Figure S1: ^1^H-NMR spectrum of curcumin, Figure S2: ^1^H-NMR spectrum of curcumin diethyl disuccinate, Figure S3: Mass spectrum of curcumin, Figure S4: Mass spectrum of curcumin diethyl disuccinate, Figure S5: HPLC chromatogram of curcumin detected at 400 nm, Figure S6: HPLC chromatogram of curcumin diethyl disuccinate detected at 400 nm, Figure S7: Powder X-ray diffraction spectrum of curcumin, Figure S8: Powder X-ray diffraction spectrum of CurDD, Figure S9: Particle size distribution analysis by laser diffraction of CurDD, Figure S10: Particle size distribution analysis by laser diffraction of curcumin.

## References

[1] Nonn L., Duong D., Peehl D.M. (2007). Chemopreventive anti-inflammatory activities of curcumin and other phytochemicals mediated by MAP kinase phosphatase-5 in prostate cells. Carcinogenesis.

[2] Basnet P., Skalko-Basnet N. (2011). Curcumin: An anti-inflammatory molecule from a curry spice on the path to cancer treatment. Molecules.

[3] Lee J.C., Kinniry P.A., Arguiri E., Serota M., Kanterakis S., Chatterjee S., Solomides C.C., Javvadi P., Koumenis C., Cengel K.A. (2010). Dietary curcumin increases antioxidant defenses in lung, ameliorates radiation-induced pulmonary fibrosis, and improves survival in mice. Radiat. Res..

[4] Yoysungnoen P., Wirachwong P., Changtam C., Suksamrarn A., Patumraj S. (2008). Anti-cancer and anti-angiogenic effects of curcumin and tetrahydrocurcumin on implanted hepatocellular carcinoma in nude mice. World J. Gastroenterol..

[5] Allegra A., Innao V., Russo S., Gerace D., Alonci A., Musolino C. (2017). Anticancer activity of curcumin and its analogues: Preclinical and clinical studies. Cancer Investig..

[6] Karunagaran D., Rashmi R., Kumar T.R.S. (2005). Induction of apoptosis by curcumin and its implications for cancer therapy. Curr. Cancer Drug Targets.

[7] Reuter S., Eifes S., Dicato M., Aggarwal B.B., Diederich M. (2008). Modulation of anti-apoptotic and survival pathways by curcumin as a strategy to induce apoptosis in cancer cells. Biochem. Pharmacol..

[8] Ravindran J., Prasad S., Aggarwal B.B. (2009). Curcumin and cancer cells: How many ways can curry kill tumor cells selectively?. AAPS J..

[9] Mortezaee K., Salehi E., Mirtavoos-mahyari H., Motevaseli E., Najafi M., Farhood B., Rosengren R.J., Sahebkar A. (2019). Mechanisms of apoptosis modulation by curcumin: Implications for cancer therapy. J. Cell. Physiol..

[10] Ratnatilaka Na Bhuket P., El-Magboub A., Haworth I.S., Rojsitthisak P. (2017). Enhancement of curcumin bioavailability via the prodrug approach: Challenges and prospects. Eur. J. Drug Metab. Pharmacokinet..

[11] Wichitnithad W., Nimmannit U., Wacharasindhu S., Rojsitthisak P. (2011). Synthesis, characterization and biological evaluation of succinate prodrugs of curcuminoids for colon cancer treatment. Molecules.

[12] Wongsrisakul J., Wichitnithad W., Rojsitthisak P., Towiwat P. (2010). Antinociceptive effects of curcumin diethyl disuccinate in animal models. J. Health Res..

[13] Bangphumi K., Kittiviriyakul C., Towiwat P., Rojsitthisak P., Khemawoot P. (2016). Pharmacokinetics of curcumin diethyl disuccinate, a prodrug of curcumin, in Wistar rats. Eur. J. Drug Metab. Pharmacokinet..

[14] Ratnatilaka Na Bhuket P., Niwattisaiwong N., Limpikirati P., Khemawoot P., Towiwat P., Ongpipattanakul B., Rojsitthisak P. (2016). Simultaneous determination of curcumin diethyl disuccinate and its active metabolite curcumin in rat plasma by LC–MS/MS: Application of esterase inhibitors in the stabilization of an ester-containing prodrug. J. Chromatogr. B.

[15] Ratnatilaka Na Bhuket P., Jithavech P., Ongpipattanakul B., Rojsitthisak P. (2019). Interspecies differences in stability kinetics and plasma esterases involved in hydrolytic activation of curcumin diethyl disuccinate, a prodrug of curcumin. RSC Adv..

[16] Aravalli R.N., Cressman E.N.K., Steer C.J. (2013). Cellular and molecular mechanisms of hepatocellular carcinoma: An update. Arch. Toxicol..

[17] Chitapanarux T., Phornphutkul K. (2015). Risk factors for the development of hepatocellular carcinoma in Thailand. J. Clin. Transl. Hepatol..

[18] Cabibbo G., Craxi A. (2010). Epidemiology, risk factors and surveillance of hepatocellular carcinoma. Eur. Rev. Med. Pharmacol. Sci..

[19] Srisuttee R., Koh S.S., Park E.H., Cho I.R., Min H.J., Jhun B.H., Yu D.Y., Park S., Park D.Y., Lee M.O. (2011). Up-regulation of Foxo4 mediated by hepatitis B virus X protein confers resistance to oxidative stress-induced cell death. Inter. J. Mol. Med..

[20] Wang M., Ruan Y., Chen Q., Li S., Wang Q., Cai J. (2011). Curcumin induced HepG2 cell apoptosis-associated mitochondrial membrane potential and intracellular free Ca^2+^ concentration. Eur. J. Pharmacol..

[21] Shoji M., Nakagawa K., Watanabe A., Tsuduki T., Yamada T., Kuwahara S., Kimura F., Miyazawa T. (2014). Comparison of the effects of curcumin and curcumin glucuronide in human hepatocellular carcinoma HepG2 cells. Food Chem..

[22] Yoysungnoen P., Wirachwong P., Bhattarakosol P., Niimi H., Patumraj S. (2005). Antiangiogenic activity of curcumin in hepatocellular carcinoma cells implanted nude mice. Clin. Hemorheol. Microcirc..

[23] OECD (1995). Test No. 105: Water Solubility.

[24] Muangnoi C., Jithavech P., Ratnatilaka Na Bhuket P., Supasena W., Wichitnithad W., Towiwat P., Niwattisaiwong N., Haworth I., Rojsitthisak P. (2018). A curcumin-diglutaric acid conjugated prodrug with improved water solubility and antinociceptive properties compared to curcumin. Biosci. Biotechnol. Biochem..

[25] OECD (2004). Test No. 117: Partition Coefficient (n-Octanol/Water), HPLC Method.

[26] Harris R.E. (2009). Cyclooxygenase-2 (cox-2) blockade in the chemoprevention of cancers of the colon, breast, prostate, and lung. Inflammopharmacology.

[27] Yoysungnoen P., Wirachwong P., Bhattarakosol P., Niimi H., Patumraj S. (2006). Effects of curcumin on tumor angiogenesis and biomarkers, COX-2 and VEGF, in hepatocellular carcinoma cell-implanted nude mice. Clin. Hemorheol. Microcirc..

[28] Olivera A., Moore T.W., Hu F., Brown A.P., Sun A., Liotta D.C., Snyder J.P., Yoon Y., Shim H., Marcus A.I. (2012). Inhibition of the NF-κB signaling pathway by the curcumin analog, 3,5-Bis(2-pyridinylmethylidene)-4-piperidone (EF31): Anti-inflammatory and anti-cancer properties. Int. Immunopharmacol..

[29] Zhou Y., Zhang T., Wang X., Wei X., Chen Y., Guo L., Zhang J., Wang C. (2015). Curcumin modulates macrophage polarization through the inhibition of the toll-like receptor 4 expression and its signaling pathways. Cell. Physiol. Biochem..

