# Supplementary material for: Scale-Up Synthesis and In Vivo Anti-Tumor Activity of Curcumin Diethyl Disuccinate, an Ester Prodrug of Curcumin, in HepG2-Xenograft Mice
Source: Pharmaceutics. 2019 Aug 1;11(8):373. doi: 10.3390/pharmaceutics11080373 (PMC6723761; doi:10.3390/pharmaceutics11080373)
Supplement: Supplementary file 1 [file pharmaceutics-11-00373-s001.pdf]

pharmaceutics

MDPI

# Supplementary Materials: Scale-Up Synthesis and In Vivo Anti-Tumor Activity of Curcumin Diethyl Disuccinate, an Ester Prodrug of Curcumin, in HepG2-Xenograft Mice

**Chawanphat Muangnoi, Pahweenvaj Ratnatilaka Na Bhuket, Ponsiree Jithavech, Wisut Wichitnithad, Onsiri Srikun, Chakkrapan Nerungsi and Suthiluk Patumraj and Pornchai Rojsitthisak**

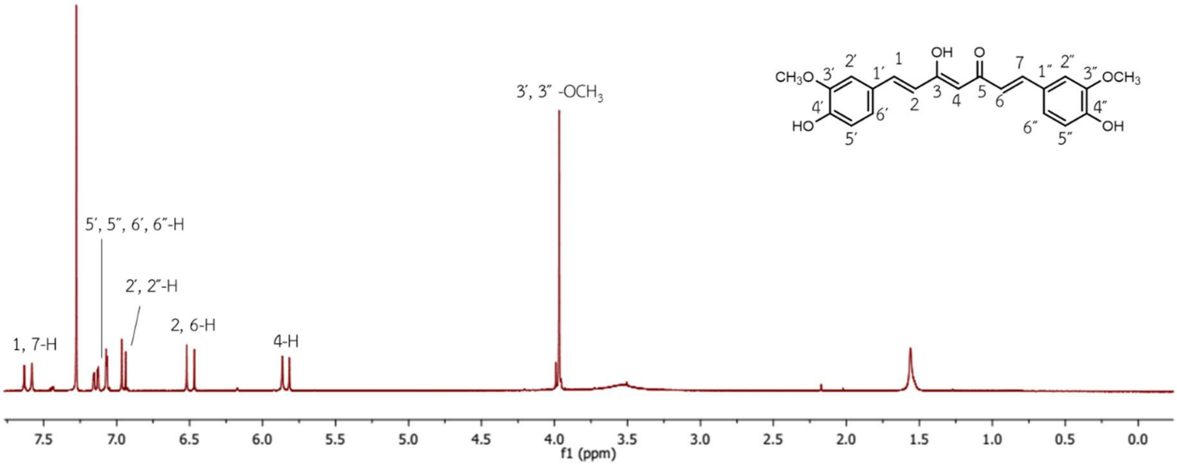


**Figure S1.** $^{1}$H-NMR spectrum of curcumin.

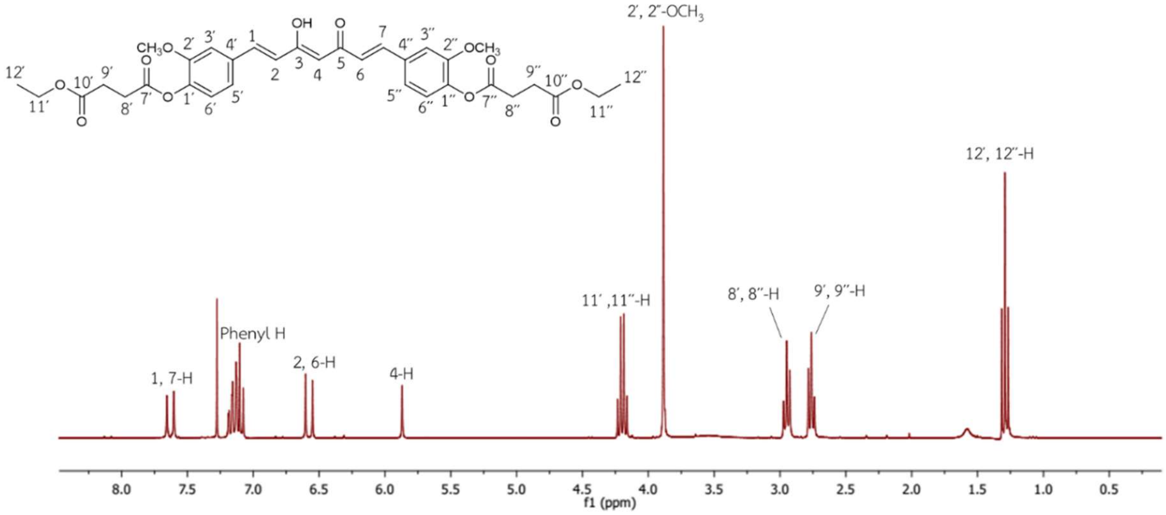


**Figure S2.** $^{1}$H-NMR spectrum of curcumin diethyl disuccinate.

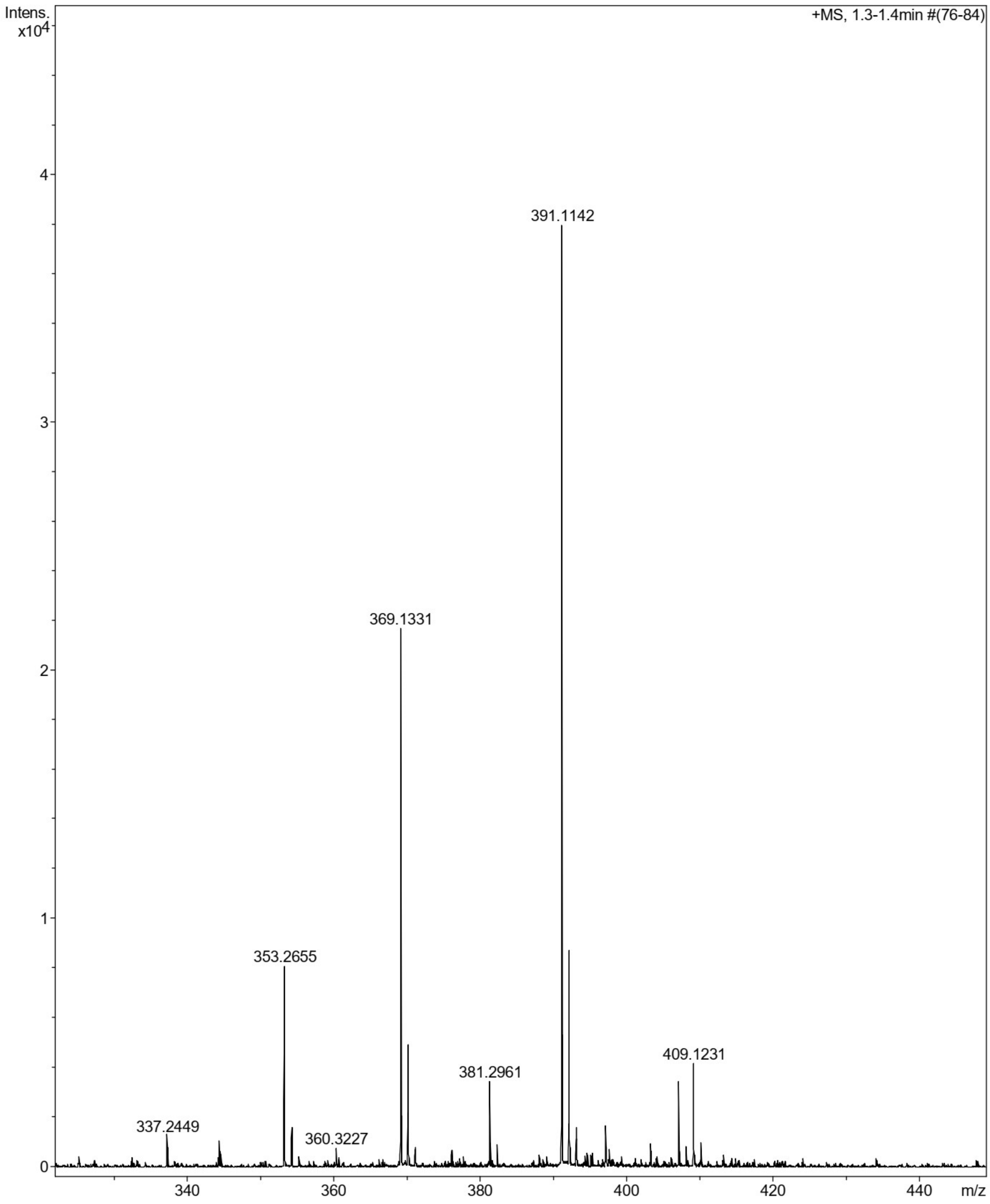


**Figure S3.** Mass spectrum of curcumin.

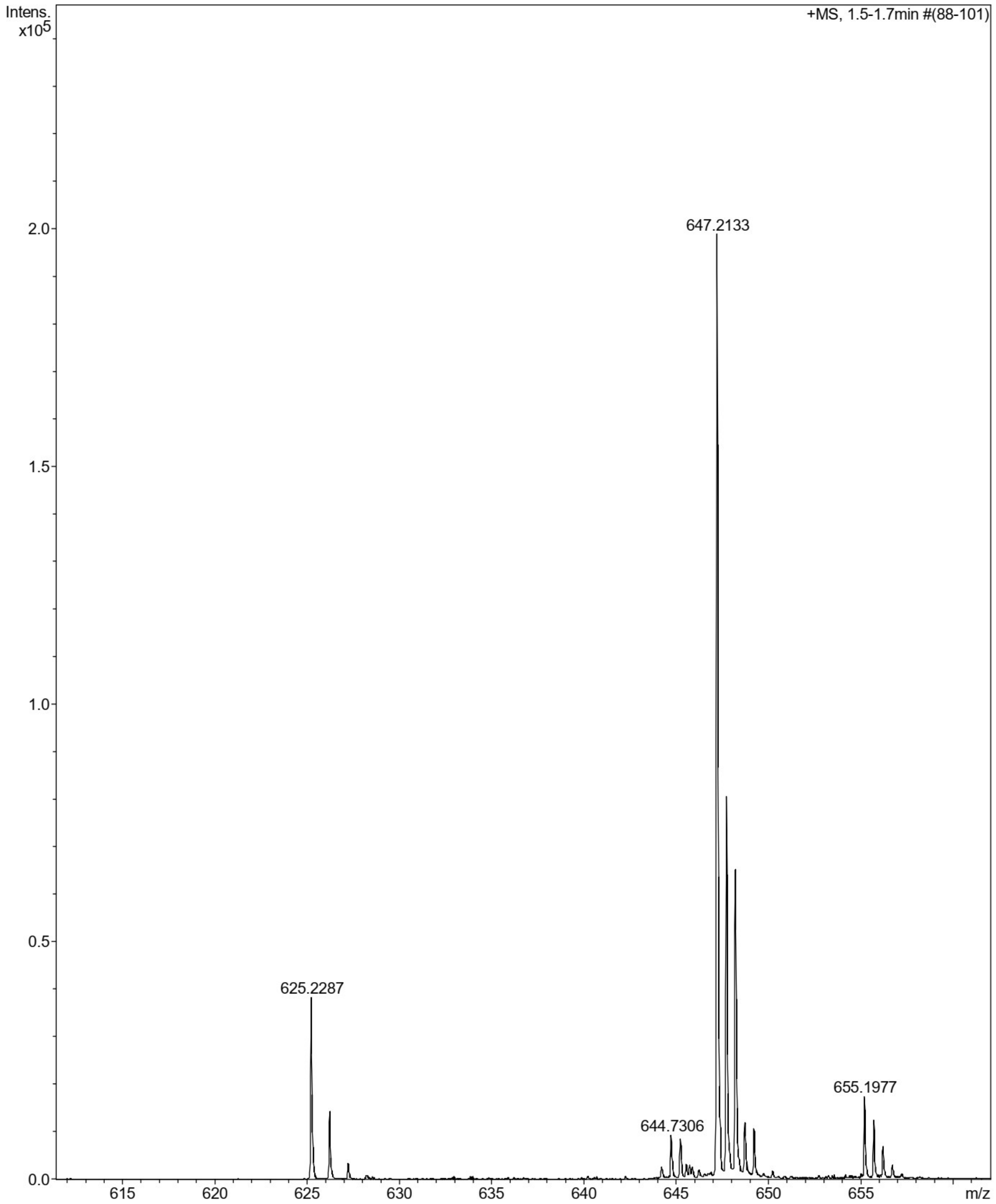


**Figure S4.** Mass spectrum of curcumin diethyl disuccinate.

## Determination of chromatographic purity of the prepared curcumin and CurDD

The prepared curcumin and CurDD were injected into an ultra-high performance liquid chromatographic system to determine chromatographic purity. The chromatographic conditions for the assay were described below.

| | |
|---|---|
| Mobile phase<br>A = 0.2 % Formic acid in water<br>B = Acetonitrile | Initial (0 min) − A:B = 55:45<br>2 min − A:B = 50:50<br>2.5 min − A:B = 50:50<br>5.0 min − A:B = 25:75<br>5.5 min − A:B = 55:45<br>7 min − A:B = 55:45 |
| Column | Acquity UPLC BEC C18 (50 mm x 2.1 mm, 1.7 µm) |
| Flow rate | 0.5 mL/min |
| Column oven temperature | 35 °C |
| Injection volume | 2 µL |
| Detection wavelength | λ = 400 nm for CurDD, 425 nm for curcumin |

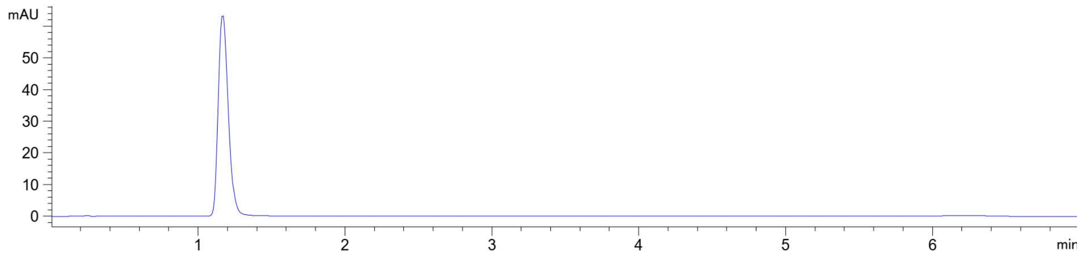


**Figure S5.** UHPLC chromatogram of curcumin.

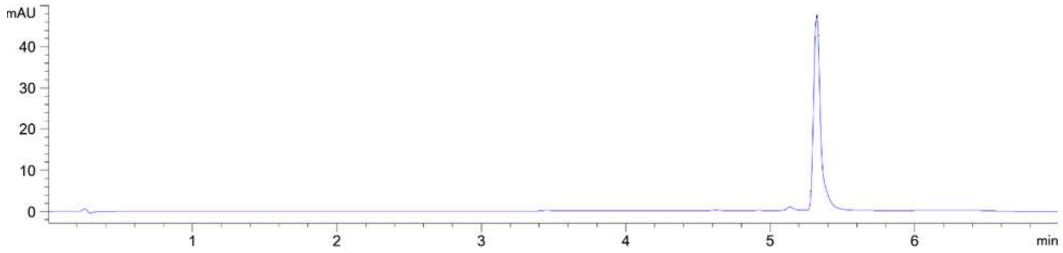


**Figure S6.** UHPLC chromatogram of curcumin diethyl disuccinate.

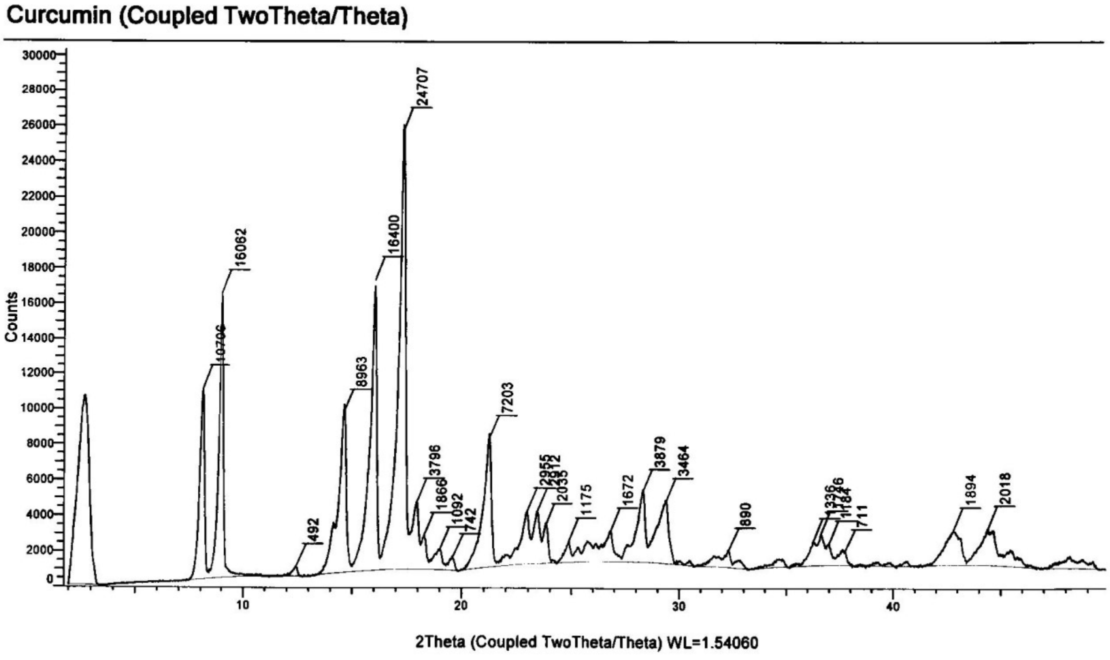


**Figure S7.** Powder X-ray diffraction spectrum of curcumin.

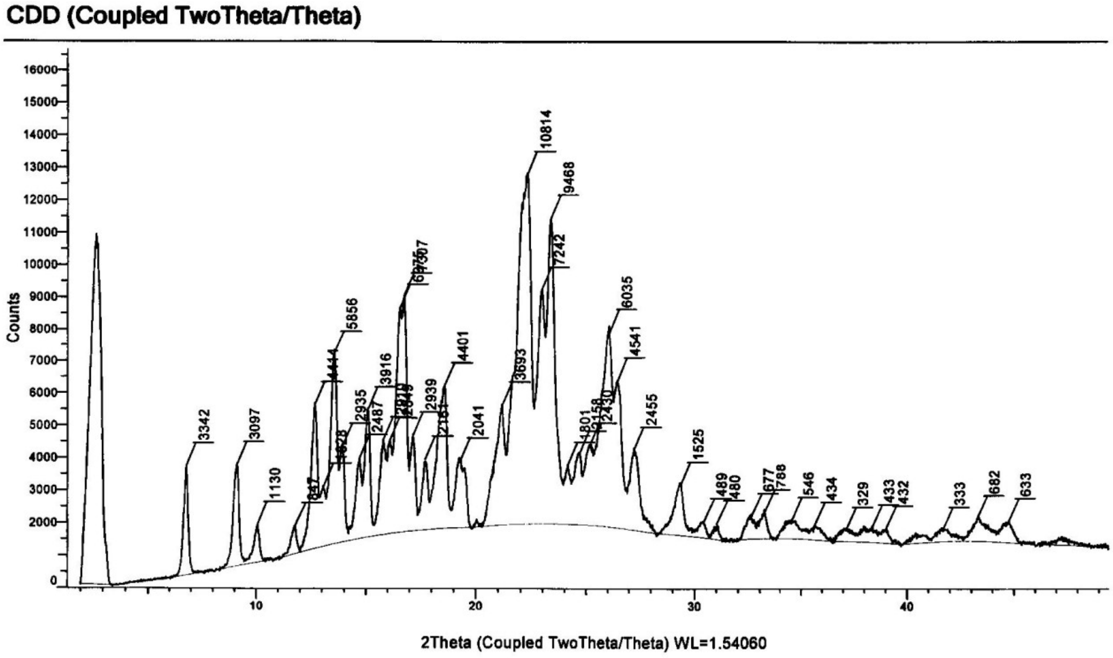


**Figure S8.** Powder X-ray diffraction spectrum of CurDD.

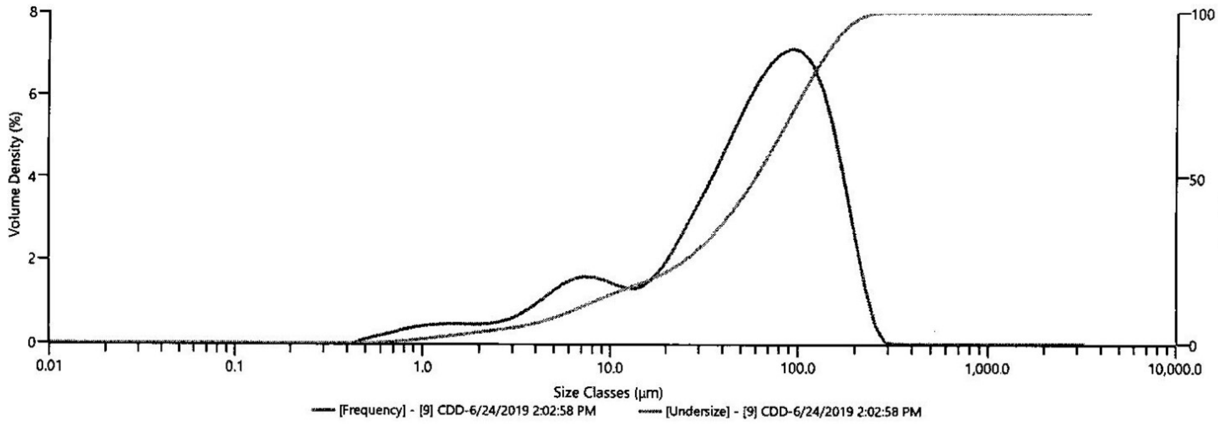


**Figure S9.** Particle size distribution analysis by laser diffraction of CurDD.

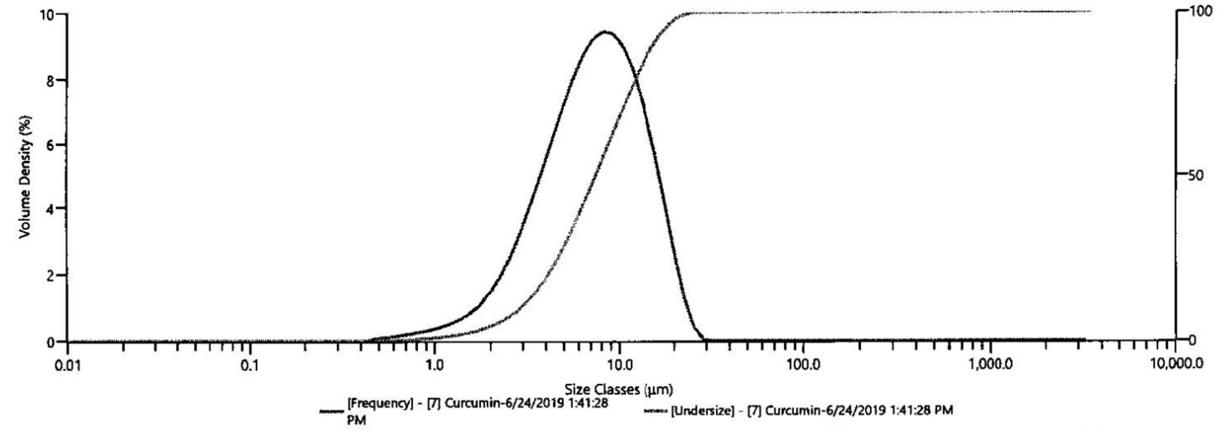


**Figure S10.** Particle size distribution analysis by laser diffraction of curcumin.
